# Long-term therapeutic effect of percutaneous kyphoplasty combined with & without back muscle rehabilitation exercise in elderly patients. A comparative study

**DOI:** 10.12669/pjms.38.6.5873

**Published:** 2022

**Authors:** Jun Jin, Weihui Shen

**Affiliations:** 1 Jun Jin, Department of General Medicine, Huzhou Cent Hospital, Affiliated Cent Hospital HuZhou University, Huzhou, Zhejiang Province 313000, P.R. China; 2 Weihui Shen, Department of Orthopedics, Huzhou Cent Hospital, Affiliated Cent Hospital HuZhou University, Huzhou, Zhejiang Province 313000, P.R. China

**Keywords:** Osteoporotic, Lumbar spine fracture, Percutaneous kyphoplasty, Back muscle rehabilitation exercise, Pain, Lumbar function

## Abstract

**Objectives:**

To know the long-term therapeutic effects and the pain improvement after percutaneous kyphoplasty (PKP) combined with and without back muscle rehabilitation exercises in elderly patients with osteoporotic lumbar compression fractures.

**Methods:**

We performed a retrospective analysis using records of elderly patients with osteoporotic lumbar compression fracture treated in our hospital from June 2019 to June 2020. We extracted relevant hospitalization treatment and record of follow-up data after discharge from 45 patients treated with PKP (Group-I) and 56 treated with PKP combined with back muscle rehabilitation exercises (Group-II). We compared the total effective lumbar function rates (number of effective and perfectly effective treatments/total number of patients) and inprovement in pain of the two treatment schemes.

**Results:**

The total efficacy in the group receiving treatment in Group-II was 96.43% higher than that of the patients receiving treatment in Group-I (84.44%; P<0.05). The pain VAS scores at one, six and 12 months in patients receiving treatment in Group-II were lower than those patients receiving treatment in Group-I (P<0.05). A year after the operation, the Oswestry disability index (ODI) scores in patients receiving treatment in Group-II were lower than those patients receiving treatment in Group-I (P<0.05). Whereas Japan Orthopaedic Association (JOA) scores were higher in the patient’s receiving treatment in Group-II as compared to Group-I (P<0.05).

**Conclusion:**

After elderly patients were treated with PKP combined with back muscle rehabilitation exercise of lumbar and dorsal muscle function, the curative effect was significantly improved, the pain was reduced and the lumbar function was significantly improved.

## INTRODUCTION

Osteoporotic lumbar spine fractures are common in the elderly population, that may cause spinal function, digestive, and respiratory problems. These fractures have become common causes of death in the elderly.^1^ The treatment strategies for these fractures comprise conservative and surgical treatments. Conservative treatment requires long-term bed rest, which may aggravate osteoporosis and lead to complications such as pneumonia, venous thrombosis, and pain.^2^,^3^

Percutaneous kyphoplasty (PKP) is a common minimally invasive option for the treatment of spinal fractures, that gives little trauma, achieves rapid pain relief, restoration of vertebral height and fixed collapsed vertebral body. However, patients retain some degree of kyphosis and soft tissue damage after the operation, which cause spine instability, pain, movement dysfunctions and other symptoms that reduce the treatment’s long-term effectivity.^4^ Therefore, it is necessary to constantly emphasize the postoperative rehabilitation exercise, commonly used clinical exercises including Baduanjin, Pilates, Taijiquan, and others, which need lumbar flexion, extension, torsion and other activities that complicate their applicability in these cases.^5^ Back muscle rehabilitation exercises can effectively enhance the function of lumbar and dorsal muscles, maintain the internal and external spine stability, adjust the spine to the mechanical changes, relief the lumbar pain, and improve the lumbar function.^6^,^7^ Our hospital has adopted the practice of prescribing back muscle rehabilitation exercises to patients with painful osteoporotic lumbar spine fractures after PKP. We designed this study to retrospectively analyze the clinical data of 101 patients treated between June 2019 and June 2020 to verify the effects of this strategy.

## METHODS

We analyzed data from 101 elderly patients (47 men and 54 women) with osteoporotic lumbar spine fractures treated in our hospital between June 2019 and June 2020. The Medical Ethics Committee of our college approved the study (Approval number: HZ210612, Date: 2021-06-13).

### Inclusion criteria:

• Meet the diagnostic criteria in the guidelines for the diagnosis and treatment of osteoporotic fractures.^8^
• Grade I~III osteoporotic vertebral compression fracture
• Present single-segment vertebral lesions.
• Be more than 60 years old.
• Have a complete medical history.

### Exclusion criteria:

• Present serious basic diseases, organ dysfunctions or malignant tumors.
• Present a multi-segmented vertebral fracture.
• Present a spinal cord injury, or lower limb neurological symptoms.
• Occurrence of vertebral body re-fracture and bone cement leakage during the operation.
• Present cognitive, mental, or language communication disorders.

### PKP treatment

Each patient was assisted to lie prone on the spinal operating frame in the operating room before performing a C-arm X-ray machine examination to observe the condition of the diseased vertebrae, mark the pedicle position, disinfect the skin, and lay a surgical towel surrounding the incision area. Local infiltration anesthesia (1% lidocaine) was administered. The puncture point was selected 1.5 cm near the posterior midline, and the needle was inserted at 2-cm of the lateral wall of the pedicle by tilting it 10-15°. The surgeon determined the position of the open needle by visualizing it using the C-arm X-ray machine. After entering the upper part of the pedicle, the surgeon advanced the needle slowly. After entering the front, middle, and lower part of the vertebral body, the puncture needle was pulled out. Next, the surgeon tapped the front 1/3 of the vertebral body and inserted the guide needle. After confirming the presence of bone around the guide, the surgeon inserted the flat head round rod to exert constant pressure on the bone. With the balloon along this channel, the surgeon slowly injected iohexol into it and observed the vertebral body in the C-arm X-ray machine. The balloon was withdrawn after reaching the satisfactory height of the vertebral body. Next, the surgeon slowly injected bone cement under C-arm X-ray machine monitoring, pulled out the working cannula after heating and solidification, and dealt with the puncture port to finish the operation.^9^ PKP combined with back muscle rehabilitation exercises. Patients started their back muscle rehabilitation exercises at three to five days postoperation when patients’ walking ability was regained:

### Five-point support

The patients were guided to lie on their back, bend their hips and knees, select five support points for their feet, elbows and head, and then raise their waist at a uniform slow speed; keeping their thighs and abdomen in a unified plane for at least 30 seconds; and then lay their waist back at a uniform speed.^10^ It was initiated at a frequency of 10 repetitions per group, and three groups a day (morning, noon and night), which could be promoted into 15 repetitions per group one week later.

### One point support training (swallow exercise)

The patients were guided to lie on their abdomen, hold their hands behind their back, straighten their legs, inhale deeply, and lift their head, upper, and lower limbs. During this process, the knee and elbow joints were extended in the shape of flying swallows. The position was maintained for five seconds, then the patients were to exhale deeply, restore their supine position, and relax for five seconds Three days to two weeks after the above training, patients were asked to perform 10 to 15 repetitions of each exercise, three times per day. From two weeks to six months after operation, each exercise was to be repeated 20 to 30 times per round, three times per day, without an upper limit.^11^ Patients were encouraged to exercise as much as possible avoiding involuntary fatigue.

We selected a visual analogue scale (VAS) score to evaluate preoperative pain; patients selected a score on a 10-score scale where 10 was the most intense pain.^12^ In preoperative lumbar function we calculated the Oswestry disability index (ODI) and Japan Orthopaedic Association (JOA) scores to assess the lumbar function of patients. The ODI includes 10 questions, each with six options scored from 0 to 5. We used the following formula to calculate the total score:

ODI = (actual score/5×number of questions answered) ×100%

The higher the score, the more serious the dysfunction.^13^ The JOA score includes subjective symptoms (9 points), clinical signs (6 points), limited daily activities (14 points), and bladder function (- 6 ~ 0 points). The lower the score, the more serious the dysfunction.^14^ We assessed the pain intensity of patients at one, six and 12 months after the operation during outpatient service, telephone or Wechat follow-ups. We evaluated the lumbar function and total efficacy one year after the operation. We used the following definitions to assess the total efficacy:^15^ Patients with completely healed fractures, without low back pain, and completely restored daily life had a perfectly effective treatment; patients with fracture healing, slight low back pain, and basic recovery of daily life had an effective treatment; and, patients with a healed fracture, with low back pain, and restricted lumbar activity, or with a condition worse than that before treatment had an ineffective treatment. Total effective rate was calculated with the formula: Total effective rate = (number of effective and perfectly effective treatments) / total number of patients.

### Statistical Analysis

We used Spss22.0 to process the data. We represented counting data as numbers and percentages [n(%)] and performed *x*^2^ tests with them, and we expressed measurement data as means and standard deviations (*x̅*±s) and performed t-tests with them. We considered all P < 0.05 as statistically significant.

## RESULTS

A total of 101 patients met the inclusion criteria (47 men and 54 women). Their age ranged from 61 to 78 years, with an average of 69.12±4.97 years. The course of disease was 3 to 12 days (average, 8.31±2.08 days). Of the 101 patients, 45 were treated with PKP and 56 with PKP combined with back muscle rehabilitation exercises. We found similar basic information between the two groups (P>0.05; Table-I).

**Table I:** Comparison of general information between the two groups.

Group	n	Gender (M/F)	Age (years)	Pain grade			Lumbar fracture site		Course of disease (days)

				I	II	III	Upper lumbar spine	Lower lumbar spine	
Group-I	45	22/23	68.55±5.21	14	22	9	23	22	8.53±2.01
Group-II	56	25/31	69.57±4.77	16	32	8	23	33	8.12±2.12
x^2^/t	-	0.181	1.02	0.856			1.014		0.982
P	-	0.671	0.31	0.652			0.314		0.326

The total efficacy of the patients receiving treatment in Group-II was 96.43%, as compared to patients in Group-I which was 84.44% (P<0.05; Table-II). Before the operation, VAS scores between the two groups were similar (P>0.05). At one, six and 12 months after the operation, the VAS scores of the two groups decreased, but those of the patient’s receiving treatment in Group-II were lower than the others (P<0.05; Table-III). Before operation, the ODI and JOA scores between the two groups were similar (P>0.05). A year after operation, the ODI scores decreased in both groups, but were lower in the patient’s receiving treatment in Group-II (P<0.05), while the JOA scores increased, but were higher in the patient’s receiving treatment in Group-II (P<0.05; Table-IV).

**Table II:** Comparison of the total effective rates between the two groups [n(%)].

Group	n	Curative effect			Total effectivity rate

		Markedly effective	Efficient	Invalid	
Group-I	45	11 (24.44)	27 (60.00)	7 (15.56)	38 (84.44)
Group-II	56	32 (57.14)	22 (39.29)	2 (3.57)	54 (96.43)
x^2^	-	-	-	-	4.415
P	-	-	-	-	0.036

**Table III:** Comparison of pain VAS scores between the two groups (*x̅*±s, points).

Group (n)	Preoperative	1 month after operation	6 months after operation	12 months after operation
Group-I (n=45)	7.87±1.23	4.02±1.07^a^	3.08±0.63^a^	1.75±0.43^a^
Group-II (n=56)	7.89±1.27	3.41±0.78^a^	1.87±0.54^a^	1.28±0.45^a^
t	0.104	3.305	10.382	5.256
P	0.917	0.001	<0.001	<0.001

***Note***: Compared with this group before nursing

a *P*<0.05.

**Table IV:** Comparison of lumbar functions between the two groups (*x̅*±s).

Group (n)	ODI (%)		*t*	*P*	JOA (points)		*t*	*P*
	
	Preoperative	12 months after operation			Preoperative	12 months after operation		
Group-I (n=45)	51.29±4.22	15.33±2.88	53.788	<0.001	10.31±2.46	20.28±2.77	20.170	<0.001
Group-II (n=56)	52.19±4.09	9.35±2.45	65.549	<0.001	10.66±2.78	25.85±3.34	31.469	<0.001
t	1.093	11.251	-	-	0.660	8.950	-	-
P	0.277	<0.001	-	-	0.511	<0.001	-	-

## DISCUSSION

For patients without surgical contraindications, surgery is the first choice in clinical treatment, PKP is a commonly used surgical method, which establishes a working channel in the vertebral body, sends the balloon, expands, and then injects bone cement, which can effectively reduce and fix the fracture. 16 Chen BL et al. found that the rehabilitation effect of patients with osteoporotic compression fractures who underwent percutaneous vertebroplasty and had sufficient muscle strength to participate in training was significantly better than that of patients without back muscle exercise.^11^ Moreover, a study by Zhu et al.^17^ pointed out that after receiving PKP treatment for osteoporotic lumbar spine fractures, patients develop disuse atrophy of low back muscles if they do not engage in effective early functional exercise. In this study, we assessed PKP combined with low back muscle functional rehabilitation exercise for elderly patients with osteoporotic lumbar spine fractures. Our results show that the total efficacy of treatment in patients with back muscle exercise after PKP was significantly better than that of patients without back muscle exercise. and the degree of postoperative pain was lower, which is consistent with the research results of Wei P and others^18^ found that the scheme of PKP combined with back muscle rehabilitation exercises can improve the curative effects of the operation alone and promote pain relief in elderly patients with osteoporotic lumbar spine fractures. The main factors causing pain in osteoporotic lumbar spine fractures include the bone stimulation of the periosteum and the adjacent sensory nerves, the increased joint load caused by the weight transfer from the vertebral body, and a muscle microcirculation disorder caused by kyphosis.^19^ PKP restores the stability of the patient’s vertebral body, relieves the peripheral nerve pain, fixes the kyphosis deformity, and reduces the load on the facet joint and intervertebral disc. PKP provides support for the reduction and stability of the lumbar spine. The functional lumbar and back muscle exercises can further promote the stability of the lumbar spine, correct the lumbar deformity and kyphosis, enhance the strength of the lumbar and back muscles, form a strong peripheral support, correct the spinal mechanics, reduce the load on the lumbar spine, and promote the recovery of damaged tissues. Thus, the exercises improve the curative effect of PKP and reduce the degree of lumbar pain.^20^

Lumbar dysfunction is a common symptom of osteoporotic lumbar spine fracture. ODI and JOA scores are common indicators of lumbar function/dysfunction.^21^ In this study, the ODI score at 12 months after the operation in the patients in Group-II was lower than as compared to patients in Group-I (P<0.05), while the JOA score at 12 months was higher in patients in Group-II than those in Group-I (P<0.05). Chiang CH et al.^22^ studies found that the ODI scores of patients after six months were significantly higher in patients performing lumbar back muscle functional exercises than in the other patients. This suggests that the application of PKP combined with back muscle rehabilitation exercises in elderly patients with osteoporotic lumbar spine fractures helps improve their lumbar function. After PKP treatment, the vertebral fracture can be reset and stably fixed. The combined back muscle rehabilitation exercises promote skeletal muscle volume growth and blood supply increases, alleviating the edema of local muscle tissue and nerve root, minimizing disuse atrophy of the muscles, and supporting the recovery of the lumbar function.^23^ Moreover, PKP combined with back rehabilitation exercises can also restore and maintain the stability of spinal mechanics, reduce excessive lumbar flexion, improve lumbar stability, increase neuromuscular control ability, and promote the lumbar function recovery.

### Limitations of the study

It includes small cohort size (101 patients), the short follow-ups of one year after the operation, and the few observation indexes with highly subjective values, which may make our conclusions limited and biased.

## CONCLUSION

PKP combined with low back muscle functional rehabilitation exercise significantly reduced the long-term pain VAS score and promoted the improvement of lumbar function in elderly patients with osteoporotic lumbar spine fractures.

### Authors’ contributions:

**JJ:** Conceived and designed the study.

**JJ & WS:** Collected the data and performed the analysis.

**JJ:** Was involved in the writing of the manuscript and is responsible for the integrity of the study.

All authors have read and approved the final manuscript.
